# Ferulic Acid Alleviates Myocardial Ischemia Reperfusion Injury Via Upregulating AMPKα2 Expression-Mediated Ferroptosis Depression

**DOI:** 10.1097/FJC.0000000000001199

**Published:** 2021-12-22

**Authors:** Xinliang Liu, Kai Qi, Yi Gong, Xiang Long, Shuqiang Zhu, Feng Lu, Kun Lin, Jianjun Xu

**Affiliations:** Department of Cardiothoracic Surgery, The Second Affiliated Hospital of Nanchang University, Nanchang, China.

**Keywords:** ferulic acid, myocardial ischemia reperfusion injury, AMPKα2, ferroptosis

## Abstract

Ferroptosis, a recently discovered form of regulated cell death that is characterized by iron accumulation and excessive reactive oxygen species generation, has been favored by most researchers. Increasing evidence suggest that ferulic acid (FA) could exert marked effects to myocardial ischemia reperfusion (I/R) injury, although the understanding of its molecular mechanism is still limited. In our study, the myocardial I/R injury model was established to explore the relationship between I/R injury and ferroptosis. First, we successfully constructed myocardial I/R injury model with changes in ST segment, increased creatine phosphokinase, lactate dehydrogenase activities, and N-Terminal Pro Brain Natriuretic Peptide content, and a significantly larger infarct size. Then, the increased levels of the Ptgs2 mRNA, Fe^2+^ accumulation, and a decreased reduced glutathione/oxidized glutathione disulfide ratio were detected in ischemia-reperfusion-injured heart, which is highly consistent with ferroptosis. However, these effects were significantly improved after FA treatment. Based on these results, FA increased the activities of the antioxidant enzymes superoxide dismutase, catalase and glutathione peroxidase, decreased the malondialdehyde level, ameliorated the production of reactive oxygen species, and promoted the generation of adenosine triphosphate. These effects of FA are similar to those of the ferroptosis inhibitor ferrostatin-1. Upregulation of AMPKα2 and Glutathione Peroxidase 4 expression were also observed in the FA group. Compound C, a specific Adenosine 5'-monophosphate (AMP)-activated protein kinase inhibitor, significantly blocked the protective effect of FA. These findings underlined that FA inhibits ferroptosis by upregulating the expression of AMPKα2 and serves as a cardioprotective strategy.

## INTRODUCTION

Ischemia reperfusion (I/R) injury occurs in numerous human organs, including the heart, kidney, liver, and brain. In recent years, many researches have been conducted on I/R injury of various organs, and our understanding of I/R injury is becoming increasingly comprehensive. Studies have shown that apoptosis,^1^ autophagy,^2^ and necroptosis^3^ are involved in the heart injury induced by I/R. Heart I/R injury is usually closely related to the excessive production of reactive oxygen species (ROS).^4^ We can improve the heart injury induced by I/R by mitigating the production of ROS. ROS are a by-product of mitochondrial oxidative phosphorylation. The accumulation of ROS leads to oxidative stress and mitochondrial damage. Therefore, mitochondrial dysfunction is a characteristic of I/R injury.^5^ Recently, ferroptosis, a newly discovered form of iron-dependent cell death, has attracted growing attention. Moreover, it has been reported that ferroptosis is involved in rat heart I/R injury, rather than ischemic injury.^6^

Heart is an organ with a high energy demand, whose function depends on mitochondria and adenosine triphosphate (ATP).^7^ Adenosine 5'-monophosphate (AMP)-activated protein kinase (AMPK), a heterotrimeric, as a sensor of energy metabolism, plays an essential role in the regulation of cardiac ischemia and reperfusion.^8^ AMPK has been reported to inhibit ferroptosis through energy stress.^9^ So, whether AMPK exerts a protective effect on myocardial I/R injury is the focus of our study.

Ferulic acid (FA) is the major active ingredient extracted from *Angelica sinensis*.^10^ FA has the characteristics of scavenging free radicals and antioxidation. FA protects epithelial cells from oxidative damage induced by H_2_O_2_.^11^ It has been reported that FA improves myocardial I/R injury through succinate dehydrogenase-dependent antioxidant activity.^12^ Moreover, FA also activates AMPK to produce an anti-inflammatory function.^13^ Nevertheless, whether FA prevents myocardial I/R injury in rats has not been clarified.

Therefore, the purposes of the present article were to explore (1) whether ferroptosis is involved in myocardial I/R injury; (2) whether FA plays a protective role against myocardial I/R injury; (3) whether the upregulation of AMPKα2 expression contributes to the protective effect of FA on myocardial I/R injury; and (4) whether the upregulation of AMPKα2 expression inhibits ferroptosis.

## MATERIALS AND METHODS

### Materials and Animals

FA (purity >98%) was acquired from Solarbio (Shanghai, China). Ferrostatin-1 (Fer-1), a specific ferroptosis inhibitor, was obtained from Selleck (Houston, TX). 2,3,5-Triphenyltetrazolium chloride (TTC) was gained from Sigma-Aldrich (St. Louis, MO). Creatine phosphokinase (CK) and lactate dehydrogenase (LDH) activity assay kits were achieved from Jiancheng (Nanjing, China). Antibodies of anti-AMPKα2, anti-Glutathione Peroxidase 4 (GPX4), and anti-glyceraldehyde-3-phosphate dehydrogenase (GAPDH) were obtained from Cell Signaling Technology (Beverly, MA). Horseradish peroxidase-conjugated IgG was purchased from Zsbio (Beijing, China).

Male Sprague-Dawley (SD) rats that were 7–8 weeks old and weighed approximately 220–250 g, were furnished by the Animal Center of Nanchang University (Nanchang, China). All experiments were conducted obeying the rule of the US National Institutes of Health Guidelines for the Care and Use of Laboratory Animals (National Institutes of Health Publication No. 85–23, revised 1996), and permission was obtained from the Ethics Committee of the Nanchang University (No. 2020–0006, Nanchang, China).

SD rats were fostered in the laboratory with clean environment and controlled temperature of 22 to 25°C, a humidity of 50%, and under a rhythm of 12-h day and night. Rats were fed diet and water routinely.

### Experimental Design

**FIGURE 1.**
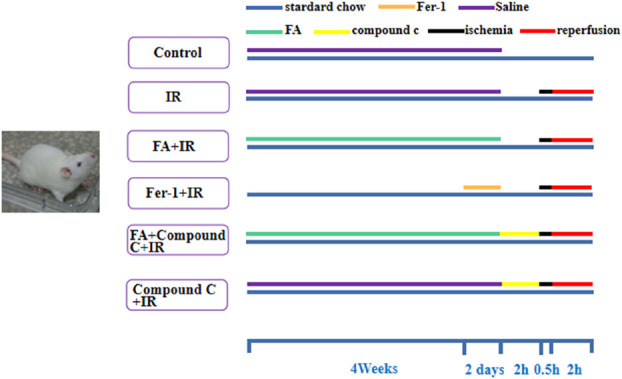
Schematic diagram of the experimental design. Forty-eight rats were randomly assigned to 6 different groups: control group, rats were fed regularly for 4 weeks; IR group, rats were fed regularly for 4 weeks; FA+IR group and FA+compound C+IR group, rats were fed regularly for 4 weeks, and 100 mg/kg FA was administered by gavage for 4 weeks; Fer-1+IR group, rats were fed regularly for 4 weeks and 1 mg/kg Fer-1 was administered by intraperitoneal injection once a day for 2 days before the establishment of the IR model; FA+compound C+IR group and compound C+IR group, rats were performed 0.2 mg/kg compound C by intravenous injection 2 hours before the establishment of the IR model; rats in the control group and A/R group were administered with an equal volume of saline. After all treatments, except for the control group, rats in other groups were subjected to 0.5 hours of ischemia and 2 hours of reperfusion. Rats in the control group underwent the same procedures without ligation of the left anterior descending artery.

As shown in Figure 1, rats were randomly distributed into 6 groups of 8 rats each: (1) control; (2) I/R; (3) FA + I/R; (4) Fer-1 + I/R; (5) FA + compound C + I/R; and (6) compound C + I/R. Rats in the FA + I/R and FA + compound C + I/R groups received 100 mg/kg FA daily for 4 weeks by oral administration.^14^ Rats in the Fer-1 + I/R group received an intraperitoneal injection of Fer-1 (1 mg/kg/d) for 2 days before I/R injury.^15^ Rats in the FA + compound C + I/R and compound C + I/R groups were administered with compound C (0.2 mg/kg, intravenous injection 2 hours before the establishment of the IR model).^16^ Rats in the control and I/R groups were treated with equal volume of saline.

### Establishment of the Myocardial I/R Model

Male SD rats were intraperitoneally injected with pentobarbital sodium at a dose of 40 mg/kg to induce anesthesia. After tracheal intubation, the rat was connected to a ventilator (inspiratory to expiratory ratio 3:1, tidal volume 2 mL, frequency 50 breaths/min). The 4 limbs of rats were fixed, and then an electrocardiogram was monitored by the standard lead II ECG. The pectoralis major muscle was dissected layer-by-layer with hemostatic forceps after skin preparation on the left side of sternum 3–4 ribs to expose the left atrial area of the heart. The pericardium was cut with ocular forceps to locate the left anterior descending branch. The left anterior descending branch was ligated with 6–0 sutures. Myocardial ischemia was caused by polyethylene plastic tubes of appropriate size to compress and occlude the coronary artery. The rats were subjected to 30 minutes of ischemia, and the plastic tube was removed to restore blood perfusion. During reperfusion for 2 hours,^17^ the thoracic cavity was clamped to reduce interference from nonexperimental factors. In the control group, the coronary artery was not ligated after the thread was punctured, and the other procedures were the same. Heart tissue and serum were gathered for subsequent studies.

### Criteria for the Successful Establishment of Myocardial I/R Model

The ECG was observed. The success of ligation was marked by an elevation in the ST segment for more than 15 minutes and cyanosis of the epicardium at the distal end of the suture. After the plastic tube was removed, the ST segment gradually returned to baseline levels, and the color of the left ventricle returned to normal.

### Determination of CK Activities, LDH Activities, and Content of NT-proBNP

Serum was collected to detect LDH and CK activities.^18^ Commercial kits were used to measure LDH and CK activities following the instructions with the help of a microplate reader (Bio-Rad 680, Hercules, CA). Serum for examination of the N-terminal pro brain natriuretic peptide (NT-proBNP). Rat NT-proBNP Elisa Kit was used to measure NT-proBNP. This test kit applies a competition method to detect the content of NT-proBNP (Nanjing Jiancheng, China).

### Measurement of Myocardial Infarction

Hearts were removed and frozen at −80°C for 10 minutes. Then the frozen hearts were cut into 1-mm transverse sections, and incubated with 2% TTC under physiological conditions (pH 7.4) for 30 minutes at 37°C. All sections were fixed with 10% formaldehyde overnight at room temperature. Sections were photographed and evaluated quantitatively using planimetry with Image J software. The infarct size was estimated as the total ventricular area minus the cavity.^19^

### Quantitative Real-Time Polymerase Chain Reaction

TRIzol was used to extract total RNA from heart tissues. The concentration and purity of RNA were detected by spectrophotometry. Reverse transcription of RNA was performed obeying the manufacturer's instructions of the RT First Strand cDNA Synthesis Kit (Servicebio, China). Polymerase chain reaction was performed using a CFX96 Real-Time System (Bio-Rad) and SYBR Green Supermix (Bio-Rad) on the basis of instructions of the manufacturer. The fold difference in Ptgs2 mRNA gene expression was analyzed using the 2^-△△Ct^ method and was shown as the fold change relative to the GAPDH mRNA.^20^ The specificity was monitored by performing a melting curve analysis. The prime sequence of ptgs2 mRNA was forward primer (5′→3′) ATGTTCGCATTCTTTGCCCAG and reverse primer (5′→3′) TACACCTCTCCACCGATGAC.

### Analysis of Iron Concentrations and the Ratio of GSH/GSSG

After the I/R model was established, the heart tissue was collected to detect iron levels. Then, the iron content in the heart was assayed as described in the manual. The absorbance was detected at 500 nm. A linear relationship was observed between the iron level and absorbance.

Reduced glutathione (GSH) is the principal source of sulfhydryl groups in most living cells. As a crucial antioxidant, GSH has the conspicuous characteristic of preserving the appropriate redox state of sulfhydryl groups in proteins. Heart tissue homogenates were used to measure GSH levels, oxidized glutathione disulfide (GSSG) levels, and the GSH/GSSG ratio^21^ according to the manufacturer's instructions. The detection kits were purchased from Beyotime Biotechnology (Haimen, Jiangsu, China).

### Detection of Antioxidant Enzyme Activities and Lipid Peroxidation

Malondialdehyde (MDA), a by-product of lipid peroxidation, was detected by kits. The activities of endogenous antioxidant enzyme systems consisting of catalase (CAT), superoxide dismutase (SOD), and glutathione peroxidase (GSH-Px) were estimated with spectrophotometry separately. Heart tissue homogenates were prepared to detect MDA levels, CAT, SOD, and GSH-Px activities^22^ strictly according to the instructions provided with the kits (Jiancheng, Nanjing, China).

### Monitoring of Energy Stress

Heart tissue homogenates were prepared in PBS and homogenate supernatants were collected for adenosine monophosphate (AMP) and ATP assays. The AMP and ATP contents were determined using ELISA kits according to the manual. The absorbance was detected with a microplate reader at 450 nm, which was positively correlated with the factors to be measured in the sample. The result was reported as the AMP/ATP ratio.^23^

### In Situ Detection of ROS

Fresh heart tissue was harvested and fixed with 4% paraformaldehyde for 24 hours. On the secondary day, the tissue was embedded in paraffin and sectioned into 7-µm thick sections that were mounted onto glass slides.

In situ detection of ROS levels in the heart tissue was performed using an efficient fluorescent probe, dihydroethidium^24^ (DHE, BestBio, Shanghai, China). Briefly, after routine dewaxing and hydration, the paraffin sections of the heart were stained with DHE (1:500) at 37°C for 30 minutes in the dark. The DHE probe was removed and the slides were washed with PBS for 3 times. The pictures were captured with an inverted fiuorescence microscope (Olympus, Tokyo, Japan) in 4 different fields for each slide randomly.

### Western Blot Analysis

Total proteins were extracted from fresh heart tissue by homogenization in RIPA buffer containing protease inhibitors, and the protein content was quantified using a BCA protein assay kit (Thermo). The protein supernatant was collected and stored at −20°C after the tissue protein lysate was centrifuged at 12,000 rpm for 15 minutes at 4°C. Twenty micrograms of protein were loaded in a 10%–12% SDS-PAGE gel and transferred to a polyvinylidene fluoride membranes. The membranes were blocked with 5% BSA for 2 hours and then incubated with primary antibodies overnight below the temperature of 4°C. The following antibodies were used: anti-GAPDH (1:1000), anti-AMPKα (1:500), and anti-GPX4 (1:1000). On the next day, the membranes were washed and incubated with a horseradish peroxidase-conjugated secondary antibody (1:2000) for 2 hours at room temperature. The protein bands were exhibited on Image Lab software after an incubated chemiluminescence reagent. The levels of protein were visualized with the help of Image J software.

### Echocardiography Detection

Transthoracic echocardiography was performed to measure cardiac function and dimensions with VINNO 6VET Imaging System (30 MHz high-frequency transducer). The rats were placed in the supine position after being anesthetized using 10% chloral hydrate. The left ventricular long axis views were used to measure the left ventricular internal diastolic diameter and left ventricular internal dimension systole, The left ventricular ejection fraction and left ventricular fractional shortening were calculated using computer software.

### Statistical Analysis

All experimental results were exhibited as the mean ± SEM, and were tested by one-way ANOVA using SPSS Statistics 24.0 software. The least significant difference test was used for further comparisons between groups. Statistical significance was considered at *P* < 0.05.

## RESULTS

### FA Treatment Protected Rat Hearts Against I/R-Induced Damage

**FIGURE 2.**
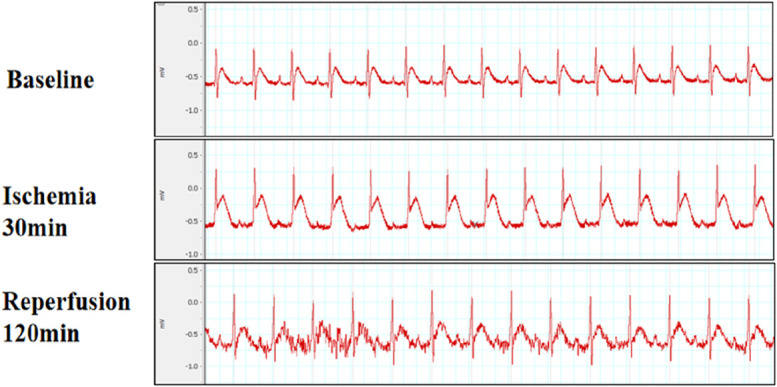
Establishment of a myocardial ischemia/reperfusion model. ECG changes were monitored with standard lead II. Typical ECG images were shown.

**FIGURE 3.**
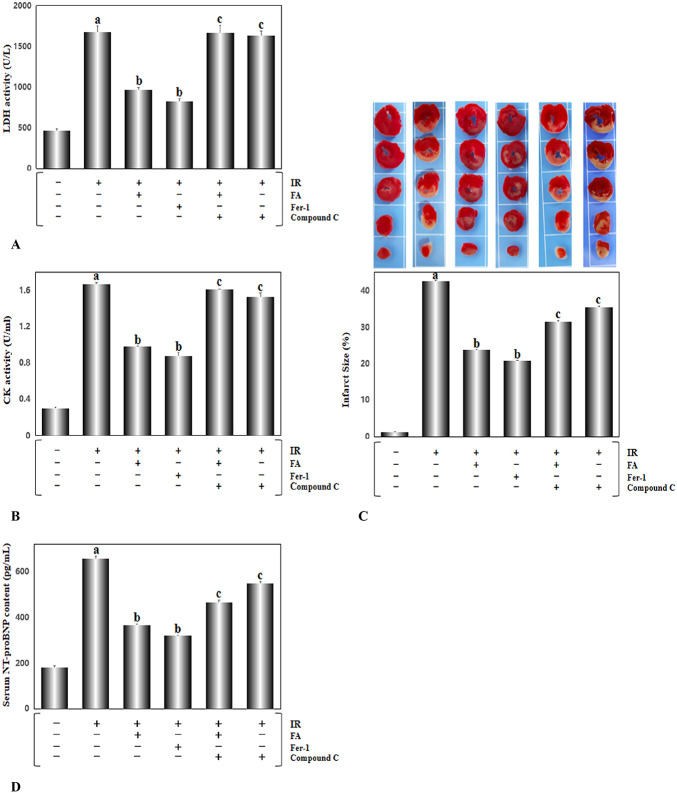

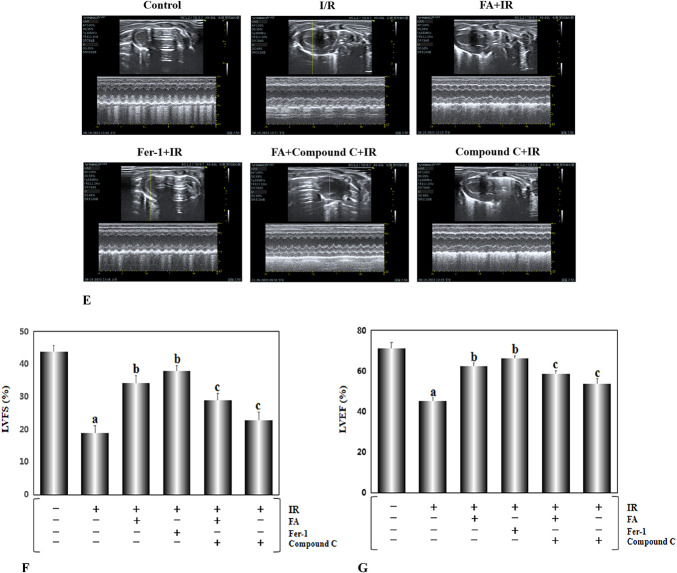
Continued

The persistent elevation of the ST segment on ECG is a marker of cardiac ischemia. As shown in Figure. 2, the ST segment ascended continuously during ischemia for 30 minutes, and gradually returned to normal levels after reperfusion, indicating that the I/R model was successfully established. Afterward, we detected the serum activities of the LDH and CK enzymes, and found that compared with the control group, the activities of LDH and CK in the I/R group increased significantly (Figs. 3A, B, *P* < 0.01). After treatment of FA and Fer-1, this process was greatly improved. However, compound C abolished the effects of FA and Fer-1. TTC staining is the gold standard indicator of myocardial ischemia.^25^ A large area of myocardial infarction was also observed in the IR group (Fig. 3C, *P* < 0.01). FA and Fer-1 significantly reduced the area of myocardial infarction induced by I/R, which was reversed by compound C. All the results showed that FA and Fer-1 significantly reduced I/R-induced damage. NT-proBNP is a biomarker in heart failure. The concentration of NT-proBNP in the I/R group was obviously increased compared with the control group. With the treatment of FA and Fer-1, this process was significantly improved compared with the I/R group. Yet, compound C could weaken the effects of FA and Fer-1 (Fig. 3D, *P* < 0.01). The LVEF and LVFS were markedly impaired after I/R (I/R). FA and Fer-1 pretreatment can improve cardiac systolic function. In addition, compound C reduced the benefit of FA and Fer-1 as shown in Figure. 3E–G (*P* < 0.01).

### FA Treatment Protected Rat Hearts from Ferroptosis Induced by I/R Damage

**FIGURE 4.**
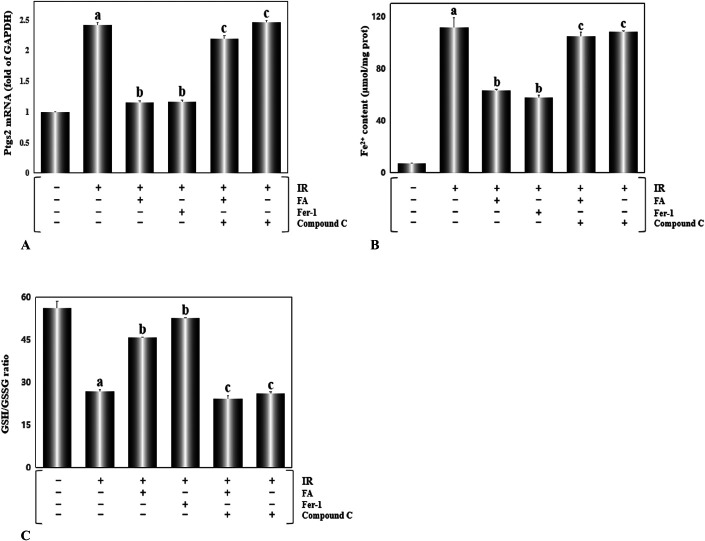
FA treatment restrained ferroptosis induced by myocardial ischemia/reperfusion injury. A, The Ptgs2 mRNA, a biomarker of ferroptosis, was assayed using real-time PCR. B, The Fe^2+^ level in the heart tissue was measured by spectrophotometry. C, The ratio of GSH/GSSG was analyzed by spectrophotometry. Results are presented as the mean ± SEM for 8 individual experiments. a, *P* < 0.01, versus the control group; b, *P* < 0.01, versus the IR group; c, *P* < 0.01, versus the FA+IR group. PCR, polymerase chain reaction

To investigate whether ferroptosis participated in I/R-induced damage. We detected 3 indicators closely related to the occurrence of ferroptosis, Ptgs2 mRNA,^26^ Fe^2+^,^15^ and GSH/GSSS ratio.^27^ As shown in Figure. 4A–C, the Ptgs2 mRNA, a marker of ferroptosis, was increased apparently in the IR group (*P* < 0.01). Obviously, enhanced Fe^2+^ levels and reduced GSH/GSSS ratios were observed in the IR group. All the above mentioned were reversed in the rats treated with FA and Fer-1. Meanwhile, both the FA+ compound C+IR group and compound C+IR group exhibited the similar results to the IR group. Based on these data, we considered the fact that the FA treatment ameliorated the damage of I/R by weakening ferroptosis.

### FA Treatment Abated Ferroptosis by Enhancing Antioxidant Enzyme Activity and Reducing MDA Levels

**FIGURE 5.**
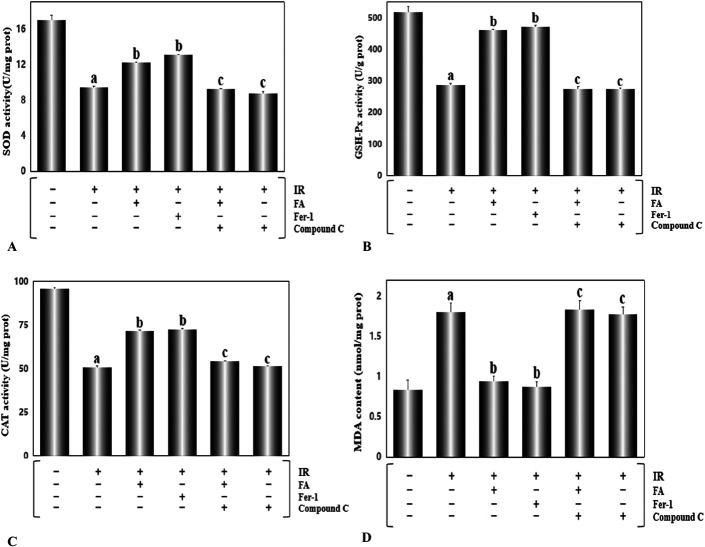
FA treatment attenuated oxidative stress and reduced MDA levels induced by myocardial ischemia/reperfusion injury. A, SOD activity in the heart tissue. B, GSH-Px activity in the heart tissue. C, CAT activity in the heart tissue. D, MDA content in the heart tissue. Data are presented as the mean ± SEM for 8 individual experiments. a, *P* < 0.01, versus the control group; b, *P* < 0.01, versus the IR group; c, *P* < 0.01, versus the FA+IR group.

**FIGURE 6.**
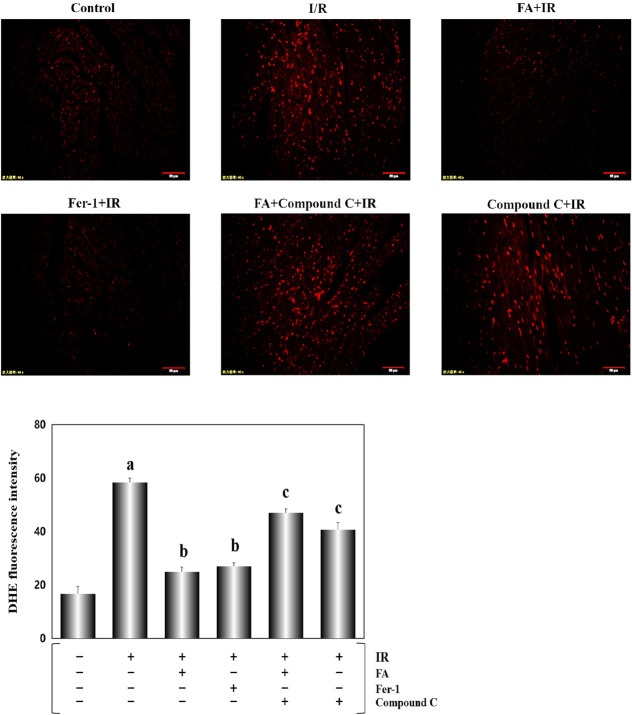
FA treatment improved in situ ROS generation in the heart tissue. A DHE fluorescent probe was used to detect in situ ROS generation in the heart tissue. Data are presented as the mean ± SEM for 8 individual experiments. a, *P* < 0.01, versus the control group; b, *P* < 0.01, versus the IR group; c, *P* < 0.01, versus the FA+IR group.

Next, we explored whether ferroptosis was associated with intracellular oxidative stress. According to studies previously published,^28^ we examined the levels of enzymes related to oxidative stress and MDA, a product of lipid peroxidation. As shown in Figure. 5A–C, compared with the control group, the IR group showed lower activities of SOD, GSH-Px, and CAT, whereas the MDA level (Fig. 5D, *P* < 0.01) was noticeably strengthened. In contrast, the hearts from rats treated with FA and Fer-1 showed a distinctly better state with significantly higher activities of antioxidant enzymes and reduced levels of MDA. Compound C prominently abolished the protective effect of FA. In addition, ROS, a stimulator of ferroptosis,^28^ were detected in situ in heart tissue sections. Consistent with results described above, FA exerted a similar inhibitory function on ROS production as Fer-1. These results indicated that I/R-induced ferroptosis was intimately related to oxidative stress, and FA substantively ameliorated this process (Fig. 6, *P* < 0.01).

### FA Treatment Lessened Ferroptosis by Promoting Energy Generation and Upregulating AMPKα2 Expression

**FIGURE 7.**
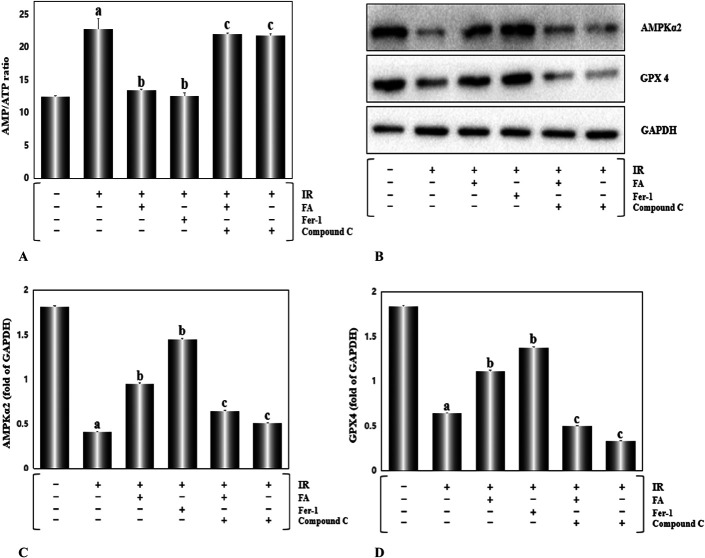
FA treatment upregulated AMPKα2 expression and inhibited ferroptosis in rat hearts. A, The ratio of AMP/ATP was assayed by ELISA. B, AMPKα2 and GPX4 expression was evaluated by Western blotting. Intake of FA for 4 weeks substantially enhanced AMPKα2 and GPX4 expression. The inhibitor of AMPK, compound C, markedly reversed the effect of FA on rat hearts. C–D, The relative expression of AMPKα2 and GPX4 in the different groups. Data are presented as fold changes relative to GAPDH levels and are shown as the mean ± SEM for 3 individual experiments. a, *P* < 0.01, versus the control group; b, *P* < 0.01, versus the IR group; c, *P* < 0.01, versus the FA+IR group.

To identify the molecular mechanism involved in I/R-induced ferroptosis, we used ELISA analysis to detect the ratio of AMP/ATP in heart tissue (Fig. 7A) which reflects the function of mitochondrial oxidative phosphorylation. A markedly increased AMP/ATP ratio was noticed in the IR group, FA+compound C+IR group and compound C+IR group. In contrast, the FA and Fer-1 treatments indicated a fine mitochondrial function, as evidenced by the decreased AMP/ATP ratio (*P* < 0.01). Consequently, our results revealed that FA exerts a positive function similar to Fer-1 on blocking ferroptosis induced by I/R damage. The above function was completely reversed in rats treated with both FA and compound C (an AMPK inhibitor) (*P* < 0.01), clarifying that FA-mediated inhibition of ferroptosis was possibly related to AMPK. To verify this hypothesis, we further investigated the expression of AMPKα2 and GPX4 (Figs. 7B–D). The positive control, Fer-1, increased AMPKα2 and GPX4 expression in rat hearts. We also found corresponding changes in FA-treated rat hearts (*P* < 0.01). Compound C and FA cotreatment can substantially weaken the protective effect of FA on IR injury, but the rats treated with compound C alone did not show a difference from the IR group. Hence, these data suggested that FA improved ferroptosis induced by I/R damage by upregulating AMPKα2 expression.

## DISCUSSION

Myocardial I/R injury is a very common clinical process in many heart diseases. Ferroptosis is a newly discovered iron-dependent cell death mode that is accompanied by a large ROS burst. Several articles have mentioned that ferroptosis is a potential therapeutic target for various cardiovascular diseases, including cardiomyopathy,^15^ myocardial infarction,^29,30^ myocardial I/R injury,^31,32^ and heart failure.^33,34^ In our study, we established a rat model of myocardial I/R injury and studied systematically, the role of ferroptosis in cardiac injury induced by cardiac I/R. We found that the activities of CK, LDH, and NT-proBNP in the I/R group were significantly increased, and a noticeably enhanced infarct size was also observed. I/R operation markedly worsens LVEF and LVFS. FA treatment significantly alleviates myocardial I/R injury, although the further experiment proved that compound C dramatically reduced cardiac function damage (Fig. 3). Moreover, several widely accepted indicators of ferroptosis (Ptgs2 mRNA, Fe^2+^ levels and GSH/GSSG ratio) were noticed to be raised remarkably in the I/R group (Fig. 4). However, these above changes were prominently rescued by treating rats with the selective ferroptosis inhibitor Fer-1, indicating that ferroptosis was indeed involved in myocardial I/R injury.^35^

FA, a polyphenolic compound, is widely distributed in Angelica, *Ligusticum chuanxiong*, and cereals.^36,37^ FA possesses strong antioxidant properties and many other pharmacological activities.^38^ FA has a strong ability to scavenge ROS and activate antioxidant enzymes, which have important functions in preventing the occurrence of cancer,^39^ cardiovascular disease,^40^ and diabetes.^41^ Our study demonstrated that augmented LDH and CK activities and the increased infarct size elicited by I/R damage, were significantly mitigated (Fig. 3) in the rats treated with FA for 4 weeks, indicating that FA reduced the cardiac injury induced by I/R. In addition, FA presented an analogous effect to the ferroptosis inhibitor Fer-1, which decreased Ptgs2 mRNA levels, attenuated Fe^2+^ accumulation, and increased the GSH/GSSG ratio (Fig. 4). We also detected the activities of several antioxidant enzymes. The results showed that both FA and Fer-1 enhanced the activities of SOD, CAT, and GSH-Px (Fig. 5). Regarding MDA, a product of lipid peroxidation, FA displayed a significant inhibitory effect. ROS, as a stimulator of ferroptosis, was detected in situ in heart tissue sections. FA eliminated the effect of I/R injury on increasing ROS production (Fig. 6). In general, FA inhibited ferroptosis elicited by I/R injury and exerted cardioprotective effects. Our findings have practical significance for the development of new uses of FA. FA-mediated inhibition of ferroptosis in myocardial I/R injury may be a feasible therapeutic approach for ischemic heart disease.

FA and resveratrol synergistically improve insulin resistance.^42^ FA mitigates arsenic-induced cardiotoxicity in rats.^43^ Wheat flour rich in FA alleviates the damage of high-glucose and high-fat diet in rats.^44^ The above mentioned functions of FA are related to the up-regulation or activation of AMPK expression. Therefore, we detected AMPKα2 expression in the heart tissues of rats after the administration of the corresponding treatment, and the AMPK inhibitor compound C was used to determine the related mechanism underlying the protective effect of FA on myocardial I/R injury. Surprisingly, the expression of AMPKα2 in the FA group was noticeably up-regulated, which was highly similar to ferroptosis inhibitor Fer-1 (Fig. 7). Nevertheless, AMPKα2 expression was obviously inhibited in the compound C treatment group. So, we considered that the cardioprotective function of FA on I/R injury was mediated by up-regulating AMPKα2 expression. It has been reported that GPX4 is a pivotal molecule regulating ferroptosis.^45,46^ We found that GPX4 expression was apparently restrained in the I/R group (Fig. 7). The FA-induced enhance in GPX4 expression was completely reversed by compound C. Mitochondria, the energy store of cells, have also been reported to be closely related to ferroptosis.^47^ In our study, we discovered that the ratio of AMP/ATP declined in the FA group, and the effect was prominently abolished by compound C. In conclusion, FA attenuated ferroptosis induced by myocardial I/R injury, and its cardioprotective effect was at least partially related to up-regulated AMPK expression to weaken ferroptosis. Further research will be conducted, and a better understanding of the mechanism of FA may provide a novel strategy for preventing myocardial reperfusion injury.

## CONCLUSIONS

In our study, we found that ferroptosis was involved myocardial I/R injury in rats. FA, a traditional Chinese medicine monomer, possesses the ability to scavenge free radicals and promote the production of free radical scavenging enzymes. Our data clarified that FA improved oxidative stress, reduced ROS overproduction, promoted GSH production, inhibited Ptgs2 mRNA and Fe^2+^ accumulation, declined LDH and CK activities, attenuated myocardial infarction, and ameliorated I/R-induced ferroptosis. It is very promising to develop FA as a protective drug for heart diseases.
